# Requirements for human natural killer cells

**DOI:** 10.1111/cpr.13588

**Published:** 2023-12-20

**Authors:** Shuaishuai Niu, Chengxiang Xia, Dehao Huang, Lei Wang, Hongbo Hu, Shuyang Yu, Ning Wu, Zhongjun Dong, Jiaxi Zhou, Jun Wu, Junying Yu, Ying Zhang, Changlin Wang, Boqiang Fu, Jiani Cao, Lingmin Liang, Lingxue Xu, Ling Chen, Qi Zhou, Aijin Ma, Tongbiao Zhao, Jie Hao, Jinyong Wang

**Affiliations:** ^1^ National Stem Cell Resource Center Institute of Zoology, Chinese Academy of Sciences Beijing China; ^2^ State Key Laboratory of Stem Cell and Reproductive Biology Institute of Zoology, Institute for Stem Cell and Regeneration, Chinese Academy of Sciences Beijing China; ^3^ Standards Committee, Chinese Society for Stem Cell Research, Chinese Society for Cell Biology Shanghai China; ^4^ Chinese Society for Stem Cell Research Shanghai China; ^5^ Beijing Institute for Stem Cell and Regenerative Medicine Beijing China; ^6^ Center for Hematology and Immunology, Cancer Center, State Key Laboratory of Biotherapy, West China Hospital Sichuan University Chengdu China; ^7^ State Key Laboratory of Animal Biotech Breeding, College of Biological Sciences China Agricultural University Beijing China; ^8^ Department of Immunology, School of Basic Medicine, Tongji Medical College Huazhong University of Science and Technology Wuhan China; ^9^ State Key Laboratory of Membrane Biology, School of Medicine and Institute for Immunology Tsinghua University Beijing China; ^10^ State Key Laboratory of Experimental Hematology, National Clinical Research Center for Blood Diseases, Haihe Laboratory of Cell Ecosystem Institute of Hematology & Blood Diseases Hospital, Chinese Academy of Medical Sciences & Peking Union Medical College Tianjin China; ^11^ University of Chinese Academy of Sciences Beijing China; ^12^ Nuwacell Biotechnology Co., Ltd Hefei City Anhui Province China; ^13^ China National Institute of Standardization China; ^14^ National Institute of Metrology Beijing China; ^15^ Savaid Medical School University of Chinese Academy of Sciences Beijing China; ^16^ Beijing Technology and Business University Beijing China

## Abstract

‘Requirements for Human Natural Killer Cells’ is the latest set of guidelines on human NK cells in China, jointly drafted and agreed upon by experts from the Standards Committee of Chinese Society for Cell Biology. This standard specifies requirements for the human natural killer (NK) cells, including the technical requirements, test methods, test regulations, instructions for use, labeling requirements, packaging requirements, storage and transportation requirements, and waste disposal requirements of NK cells. This standard is applicable for the quality control of NK cells, derived from human tissues, or differentiated/transdifferentiated from stem cells. It was originally released by the Chinese Society for Cell Biology on 30 August, 2022. We hope that the publication of these guidelines will promote institutional establishment, acceptance, and execution of proper protocols and accelerate the international standardization of human NK cells for applications.

## 1 SCOPE

This document specifies requirements for the human natural killer (NK) cells, including the technical requirements, test methods, test regulations, instructions for use, labeling requirements, packaging requirements, storage and transportation requirements, and waste disposal requirements of NK cells.

This standard is applicable for the quality control of NK cells, derived from human tissues, or differentiated/transdifferentiated from stem cells.

## 2 NORMATIVE REFERENCES

The following documents are referred to in the text in such a way that some or all of their content constitutes requirements of this document. For dated references, only the edition cited applies. For undated references, only the latest edition (including all amendments) applies.

WS 213 Diagnosis for hepatitis C.

WS 273 Diagnosis for syphilis.

WS 293 Diagnosis for HIV/AIDS.

T/CSCB 0001 General Requirements for Stem Cells.

T/CSCB 0002 Requirements for human embryonic stem cells.

Pharmacopoeia of the People's Republic of China, 2020 Edition Three.

National Guide to Clinical Laboratory Procedures, Fourth Edition.

## 3 TERMS AND DEFINITIONS

The following terms and definitions apply to this document.

### 3.1 Human natural killer cells

NK cells are isolated from human tissues or obtained by differentiation of stem cells or transdifferentiation of other cell types. It belongs to the intrinsic lymphocytes and has three functions, non‐specific killing, antibody‐dependent cellular cytotoxicity, and immune‐modulation.

### 3.2 Non‐specific killing

Non‐specific killing refers to direct killing of tumour cells or virus‐infected cells without the necessities of somatic gene rearrangement, expression of specific antigen‐recognition receptors, and pre‐sensitization.

### 3.3 Antibody‐dependent cellular cytotoxicity, ADCC

ADCC refers to the mechanism by which NK cells expressing FcγRIII (CD16) can recognize the Fc segment of antigen‐bound antibody, thus killing the antibody‐bound target cells.

### 3.4 Immunomodulation of natural killer cells

NK cells interact with other immune cells by releasing a large number of cytokines and chemokines and regulate the immune state and immune function of the body.

## 4 ABBREVIATIONS

The following abbreviations are applicable for this document.

ADCC—Antibody‐Dependent Cellular Cytotoxicity.

CFSE—Carboxyfluorescein Succinimidyl Ester.

HLA—Human Leukocyte Antigen.

NK—Natural Killer.

STR—Short Tandem Repeat.

## 5 TECHNICAL REQUIREMENTS

### 5.1 Cell morphology

NK cells are cultured in vitro in a suspended state. After activation, their volume shall become larger, and the cell shape shall become irregular.

### 5.2 Chromosome karyotype

The normal karyotype shall be 46, XX, or 46, XY.

### 5.3 Cellular markers

The immunophenotyping of NK cells described as follows:

The expressions of CD56 and CD45 shall be≥90% of the cell population, and the expression of CD16 shall be ≥10% of the cell population.

### 5.4 Cell purity

The expressions of CD3 shall be ≤5% of the cell population.

### 5.5 Cellular activity and function

NK cells shall kill the cells whose HLA‐I expression is down‐regulated or lost. After activation, NK cells shall release granzyme, perforin, and interferon. Combined antibodies can enhance the cytotoxicity of NK cells.

Note: For example, NK cells have cytolytic activity against solid tumour cells (ovarian cancer cells) and haematological tumour cells (myeloid leukaemia tumour cells) with down‐regulated expression of HLA‐I molecules.

### 5.6 Cell viability

The cell viability shall be≥70% prior to cryopreservation, and ≥ 50% after thawing but before the application.

### 5.7 Microorganisms

Cells shall be negative for fungi, bacteria, mycoplasma, chlamydia, and viruses.

### 5.8 Endotoxin

Endotoxin content shall be ≤1 EU/mL.

### 5.9 Identification of cell STR

NK cells isolated from human tissues, differentiated or transdifferentiated from stem cells shall be subjected to STR assay and typing identification, without cross‐contamination between samples.

## 6 TEST METHODS

### 6.1 Cell morphology

Observe the morphology of cells grown under 2D conditions in vitro using a microscope.

### 6.2 Chromosome karyotype

The method in the Pharmacopoeia of the People's Republic of China shall be followed.

Note: Determine the appropriate karyotype result based on the characteristics of the target sample. Normal karyotype shall be 46, XX, or 46, XY.

### 6.3 Cell markers

The method in Appendix A shall be followed.

### 6.4 Cell purity

The method in Appendix A shall be followed.

### 6.5 Cellular activity and function

The detection method for granzyme and perforin in activated NK cells shall be tested according to the method in Appendix B.

The detection method for the release of interferon in activated NK cells shall be tested according to the method in Appendix B.

The cytotoxicity assay of NK cells shall be carried out according to the method in Appendix C.

The ADCC detection of NK cells shall be tested according to the method in Appendix D.

### 6.6 Cell viability

The method ‘Cell viability test’ in the T/CSCB 0002 shall be followed.

### 6.7 Microorganisms

Fungi.

The method ‘1101 sterility test’ in the Pharmacopoeia of the People's Republic of China shall be followed.

Bacteria.

The method ‘1101 sterility test’ in the Pharmacopoeia of the People's Republic of China shall be followed.

Mycoplasma.

The method ‘3301 sterility test’ in the Pharmacopoeia of the People's Republic of China shall be followed.

Human immunodeficiency virus.

The nucleic acid test method in WS 293 shall be followed.

Hepatitis B virus.

The nucleic acid test method in the National Guide to Clinical Laboratory Procedures shall be followed.

Human T‐cell lymphotropic virus.

The nucleic acid test method in the National Guide to Clinical Laboratory Procedures shall be followed.

Human cytomegalovirus.

The nucleic acid test method in the National Guide to Clinical Laboratory Procedures shall be followed.

Hepatitis C virus.

The nucleic acid test method in WS 213 shall be followed.

Treponema pallidum.

The nucleic acid test method in WS 273 shall be followed.

### 6.8 Endotoxin

The method ‘1143 bacterial and endotoxin test’ in the Pharmacopoeia of the People's Republic of China shall be followed.

### 6.9 Cell STR identification

The method ‘Cell authentication’ in the T/CSCB 0002 shall be followed.

## 7 INSPECTION RULES

### 7.1 Batch definition

Cells produced from the same production cycle, same production line, same source, same passage, and same method are considered as the same batch.

### 7.2 Sampling method

Three smallest units of packaging shall be randomly sampled from the same batch.

### 7.3 Quality inspection and release

7.3.1 Each batch of products shall be subject to the qualified inspection before release, and inspection reports shall be attached.
7.3.2 The quality inspection items shall include all the attributes specified in 5.

### 7.4 Review inspection

Review inspection shall be performed by professional cytological testing institutions/laboratories as necessary.

### 7.5 Decision rules

7.5.1 Products that pass all requirements in 5 for the quality inspection for release are considered as qualified. Products that fail to pass one or more requirements in 5 for the quality inspection for release are considered as unqualified.
7.5.2 Products that pass all requirements in 5 for the quality review inspection are considered as qualified. Products that fail to pass one or more requirements in 5 for the review inspection are considered as unqualified.

## 8 INSTRUCTIONS FOR USAGE

The instructions for usage shall include, but not be limited to

1. Product name;

2. Passage number;

3. Cell number;

4. Production date;

5. Lot number;

6. Production organization;

7. Storage conditions;

8. Shipping conditions;

9. Contact information;

10. Operation manual;

11. Execution standard number;

12. Manufacturing address;

13. Postal code;

14. Matters that deserve attention.

Note: Upon user's requirement, endotoxin test results can be provided.

## 9 LABELS

The label shall include but not be limited to.

1. Product name;

2. Passage number;

3. Cell number;

4. Lot number;

5. Production organization;

6. Production date.

## 10 PACKAGING, STORAGE, AND TRANSPORTATION

### 10.1 Package

The appropriate materials and containers shall be selected to ensure maintenance of the primary quality attributes of NK cells.

### 10.2 Storage

10.2.1 T/CSCB 0001 shall be followed.
10.2.2 Productions shall be stored in an environment not higher than −130°C.

### 10.3 Transportation

10.3.1 T/CSCB 0001 shall be followed.
10.3.2 Cryopreserved cells shall be transported in dry ice or below‐130°C, while non‐cryopreserved cells shall be transported at 2–8°C.

## 11 WASTE DISPOSAL

The waste generated during the production and testing of human natural killer cells shall be in accordance with the waste cell management documents, strict implementation of management standards, and detailed records.

Unqualified cells, remaining discarded cells, or donations in the research and production of human retinal pigment epithelial cells shall be disposed of legally, properly, and ethically.

## AUTHOR CONTRIBUTIONS

Wang J, Hao J, Zhao T, and Ma A contributed to conception and design. Niu S, Xia C, Huang D, Wang L drafted and revised the manuscript. Hu H, Yu S, Wu N, Dong Z, Zhou J, Zhang Y, Wu J, Yu J, Wang C, Fu B, Cao J, Liang L, Xu L, Chen L and Zhou Q critically read and revised the manuscript.

## CONFLICT OF INTEREST STATEMENT

The authors declare no competing financial interests.

## Data Availability

Research data are not shared.

## APPENDIX (NORMATIVE) A DETECTION OF CELLULAR MARKER PROTEINS AND ASSESSMENT OF CELL PURITY (FLOW CYTOMETRY)

### A.1 INSTRUMENTS

A.1.1 Flow cytometry
A.1.2 Centrifuge

### A.2 REAGENT

Unless otherwise stated, all the reagents used shall be analytical grade. The water used in the experiment shall be Grade 1 water as stipulated in GB/T 6682.

A.2.1 Phosphate buffered saline (PBS): pH 7.4
A.2.2 PBS with 2% Fetal Bovine Serum (FBS) is recommended for use as a buffer to dilute or wash nucleated cells
A.2.3 Relevant antibodies mentioned in context and corresponding isotype control antibodies
A.2.4 Prepare the following solutions according to the relative requirements for flow cytometry: wash solution, and antibody dilution solution

### A.3 SAMPLE STORAGE

The wash solution and stained samples shall be stored at 2–8°C. Antibodies shall be stored according to the manufacturer's instructions.

### A.4 TESTING PROTOCOL

A.4.1 Sample preparation and antibody incubation
  Collect samples by centrifuging single‐cell suspensions at 250 g for 3 min. Discard the supernatant. Incubate the samples with the diluted antibodies or corresponding isotype control antibodies according to the manufacturer's instructions.
  A.4.2 Flow cytometry analysis
  Resuspend the samples with wash solution and then transfer the cell suspension into flow cytometry tube by filtering the samples through a mesh with 40 μm pores. Load the samples into the flow cytometer and perform testing according to the manufacturer's instruction.
A.4.2 Gating
  Gate the target population of cells based on particle size and transparency, excluding cell debris and other irrelevant particles. The gating of positive staining cells shall be determined by the fluorescence intensity using isotype controls as a reference. Both positive and negative experimental controls shall be set up for gating and the following analysis.

### A.5 ANALYSIS OF RESULTS

Analyse the results using software according to manufacturer's instructions.

## APPENDIX (NORMATIVE) B ANALYSIS OF CD107A, PERFORIN, AND GRANZYME B EXPRESSION LEVELS IN ACTIVATED NK CELLS (FLOW CYTOMETRY)

### B.1 INSTRUMENTS

B.1.1 Flow cytometry
B.1.2 Centrifuge
B.1.3 Cell culture incubator

### B.2 REAGENTS

B.2.1 PBS: pH 7.4
B.2.2 PBS with 2% FBS is recommended for use as a buffer to dilute or wash nucleated cells
B.2.3 Relevant antibodies mentioned in context and corresponding isotype control antibodies
B.2.4 Prepare the following solutions according to the relative requirements for flow cytometry: wash solution, and antibody dilution solution
B.2.5 Target cells, such as K562 cells (leukaemia cell line established from a female patient with chronic myelogenous leukaemia)

### B.3 SAMPLE STORAGE

The wash solution and stained samples shall be stored at 2–8°C. Antibodies shall be stored according to the manufacturer's instructions.

### B.4 TESTING PROTOCOL

B.4.1 Effector cell and target cell collection
  Effector NK cells and target cells, such as K562 cells, shall be collected separately. Single‐cell suspensions shall be prepared and counted.
B.4.2 Coincubation of effector cells with target cells
  NK cells were incubated with or without target cells at 1:3 effector‐to‐target cell ratio (e.g., E:T = 2 × 10^5^: 6 × 10^5^). The corresponding reagents for each experiment shall be added to each well and allowed to incubate for 1 h in an incubator with humidified atmosphere (5% CO_2_ and 37°C).
B.4.3 Flow cytometry analysis
  The expression levels of CD107a (degranulation ability), granzyme B, and perforin in NK cells shall be detected separately according to the flow cytometry assay procedure. Among them, CD107a shall be extracellular staining, and both Granzyme B and Perforin shall be intracellular staining.
B.4.4 Gating
  Gate the target population of cells based on particle size and transparency, excluding cell debris and other irrelevant particles. The gating of positive staining cells shall be determined by the fluorescence intensity using isotype controls as a reference. Both positive and negative experimental controls shall be set up for gating and the following analysis. NK cell populations shall be defined according to CD3^−^CD45^+^CD56^+^, followed by analysis of positive proportions of CD107a, granzyme B, and perforin from NK cells.

### B.5 ANALYSIS OF RESULTS

Analyse the results using software according to manufacturer's instructions.

## APPENDIX (NORMATIVE) C NK CELLS CYTOTOXICITY ASSAYS (FLOW CYTOMETRY)

### C.1 INSTRUMENTS

C.1.1 Flow cytometry
C.1.2 Centrifuge
C.1.3 Cell culture incubator

### C.2 REAGENTS

C.2.1 RPMI1640 complete medium
C.2.2 PBS solution
C.2.3 PBS with 2% FBS is recommended for use as a buffer to dilute or wash nucleated cells
C.2.4 CFSE assay kit
C.2.5 Target cells, such as K562 cells

### C.3 TESTING PROTOCOL

C.3.1 CFSE‐labelled target cells
  The target cells such as K562 cells shall be labelled by CFSE according to the instructions.
C.3.2 Coincubation of effector cells with target cells
  Effector NK cells shall be incubated with CFSE labelled target cells at 0:1/1:1/5:1/10:1 effector to target ratios, mixed thoroughly in a 96‐well culture plate, and incubated for 4 h in an incubator with humidified atmosphere (5% CO_2_ and 37°C). Then, the cells in the 96‐well culture plate shall be collected, resuspended with PBS + 2% FBS solution, and incubated with 7‐AAD staining solution at 2–8°C for 15 min protected from light according to the instructions for use, and then subjected to flow cytometry analysis.
C.3.3 Flow cytometry analysis
  Following the flow cytometry assay procedure, flow cytometry shall be used to detect the proportion of 7‐AAD‐positive cells among CFSE‐positive target cells in co‐incubated cells.
C.3.4 Gating
  Gate the target population of cells based on particle size and transparency, excluding cell debris and other irrelevant particles. The gating of positive staining cells shall be determined by the fluorescence intensity using isotype controls as a reference. Both positive and negative experimental controls shall be set up for gating and the following analysis. The percentage of 7‐AAD positive cells among the CFSE positive target cells shall be analysed.

### C.4 ANALYSIS OF RESULTS

Analyse the results using software according to manufacturer's instructions.

## APPENDIX (NORMATIVE) D ANALYSIS OF NK CELL‐MEDIATED ADCC FUNCTION (FLOW CYTOMETRY)

### D.1 INSTRUMENTS

D.1.1 Flow cytometry
D.1.2 Centrifuge
D.1.3 Cell culture incubator

### D.2 REAGENTS

D.2.1 RPMI1640 complete medium
D.2.2 Rituximab
D.2.3 CFSE assay kit
D.2.4 Target cells, such as Raji (NK cell‐resistant CD20 positive Human Burkitt's lymphoma cell line)

### D.3 TESTING PROTOCOL

D.3.1 CFSE‐labelled target cells
  The target cells such as Raji cells were labelled by CFSE according to the instructions.
D.3.2 Coincubation of effector cells with target cells
  Effector NK cells shall be incubated with CFSE labelled target cells at effector‐to‐target cell (E:T) ratios of 0:1,5:1, and 10:1, and divided into two groups per E:T ratio mixture according to the added antibodies: no antibody group and Rituximab added. The cells shall be mixed well in 96‐well culture plates and incubated for 4 h in an incubator with humidified atmosphere (5% CO_2_ and 37°C). Then, the cells in the 96‐well culture plate shall be collected, resuspended with PBS + 2% FBS solution, and incubated with 7‐AAD staining solution for 15 min at 2–8°C and protected from light according to the instructions for use, and then subjected to flow cytometry analysis.
D.3.3 Flow cytometry analysis
  Following the flow cytometry assay procedure, flow cytometry shall be used to detect the proportion of 7‐AAD‐positive cells among CFSE‐positive target cells in co‐incubated cells.
D.3.4 Gating
  Gate the target population of cells based on particle size and transparency, excluding cell debris and other irrelevant particles. The gating of positive staining cells shall be determined by the fluorescence intensity using isotype controls as a reference. Both positive and negative experimental controls shall be set up for gating and the following analysis. The percentage of 7‐AAD positive cells among the CFSE‐positive target cells was then analysed.

### D.4 ANALYSIS OF RESULTS

Analyse the results using software according to manufacturer's instructions.

